# Author Correction: Effect of *de novo* transcriptome assembly on transcript quantification

**DOI:** 10.1038/s41598-019-53955-z

**Published:** 2019-11-27

**Authors:** Ping-Han Hsieh, Yen-Jen Oyang, Chien-Yu Chen

**Affiliations:** 1 Graduate Institute of Biomedical Electronics and Bioinformatics, National Taiwan University, Taipei, 10617 Taiwan; 2 Department of Computer Science and Information Engineering, National Taiwan University, Taipei, 10617 Taiwan; 3 Department of Bio-Industrial Mechatronics Engineering, National Taiwan University, Taipei, 10617 Taiwan; 4 Genome and Systems Biology Program, National Taiwan University and Academia sinica, Taipei, 10617 Taiwan

Correction to: *Scientific Reports* 10.1038/s41598-019-44499-3, published online 05 June 2019

The Supplementary File 1 originally published with this Article contained typographical errors where ‘rnaSPAdes’ was incorrectly given as ‘rnaSPades’. Additionally, in Table S7 the column subheadings ‘Salmon’ and ‘RSEM’ were inverted. These errors have been corrected in the Supplementary File 1 that now accompanies the Article.

