# Supplementary material for: Effect of de novo transcriptome assembly on transcript quantification
Source: Sci Rep. 2019 Jun 5;9:8304. doi: 10.1038/s41598-019-44499-3 (PMC6549443; doi:10.1038/s41598-019-44499-3)
Supplement: Supplementary file 1 [file 41598_2019_44499_MOESM1_ESM.docx]

**Effect of *de novo* transcriptome assembly on transcript quantification**

Ping-Han Hsieh, Yen-Jen Oyang and Chien-Yu Chen

**Supplementary File 1**

Table S1-1

*Parameter settings used when invoking the Flux Simulator for simulated dataset*

|  | Yeast | Dog | Mouse |
| --- | --- | --- | --- |
| NB_MOLECULES | 5,000,000 | 35,000,000 | 85,000,000 |
| FRAGMENTATION | YES | YES | YES |
| FRAG_SUBTRATE | RNA | RNA | RNA |
| FRAG_METHOD | UR | UR | UR |
| RTRASCRIPTION | YES | YES | YES |
| RT_PRIMER | RH | RH | RH |
| FILTERING | YES | YES | YES |
| PCR_PROBABILITY | 0.05 | 0.05 | 0.05 |
| ERR_FILE | 76 | 76 | 76 |
| PAIRED_END | YES | YES | YES |
| READ_LENGTH | 150 | 150 | 150 |
| READ_NUMBER | 3,000,000 | 21,000,000 | 50,000,000 |

Table S1-2

*Parameter settings used when invoking the Flux Simulator for simulated (H) dataset*

|  | Yeast | Dog | Mouse |
| --- | --- | --- | --- |
| NB_MOLECULES | 5,000,000 | 35,000,000 | 85,000,000 |
| FRAGMENTATION | YES | YES | YES |
| FRAG_SUBTRATE | RNA | RNA | RNA |
| FRAG_METHOD | UR | UR | UR |
| RTRASCRIPTION | YES | YES | YES |
| RT_PRIMER | RH | RH | RH |
| FILTERING | YES | YES | YES |
| PCR_PROBABILITY | 0.05 | 0.05 | 0.05 |
| ERR_FILE | 76 | 76 | 76 |
| PAIRED_END | YES | YES | YES |
| READ_LENGTH | 150 | 150 | 150 |
| READ_NUMBER | 100,000,000 | 100,000,000 | 100,000,000 |

Table S2-1

*Sequence information of RNA-Seq reads used in this study*

|  | Experimental data | | |
| --- | --- | --- | --- |
|  | Yeast | Dog | Mouse |
| No. of fragments | 5,449,060 | 20,480,445 | 43,145,380 |
| *Max* read length | 101 | 50 | 76 |
| Sequencing depth | 118.0 | 33.2 | 44.5 |
| Insert size *mean*^*^ | 204.6 | 150.5 | 289.8 |
| Insert size *SD*^*^ | 96.3 | 53.9 | 78.1 |
| Strand specificity | Non-stranded | Non-stranded | RF-stranded |
|  | Simulated data | | |
|  | Yeast | Dog | Mouse |
| No. of fragments | 1,212,904 | 8,464,764 | 20,209,629 |
| *Max* Read length | 150 | 150 | 150 |
| Sequencing depth | 33.5 | 32.1 | 33.1 |
| Insert size *mean^*^* | 193.8 | 194.0 | 194.1 |
| Insert size *SD*^*^ | 30.2 | 30.1 | 30.1 |
| Strand specificity | Non-stranded | Non-stranded | Non-stranded |

^*^ The mean and standard deviation of the insert sizes were estimated using Burrows-Wheeler Aligner.

Table S2-2

*Sequence information of RNA-Seq reads with higher sequencing depth*

|  | Experimental (H) data | | |
| --- | --- | --- | --- |
|  | Yeast | Dog |  |
| No. of fragments | 18,008,372 | 34,653,792 |  |
| *Max* read length | 101 | 50 |  |
| Sequencing depth | 387.6 | 55.4 |  |
| Insert size *mean*^*^ | 199.3 | 143.3 |  |
| Insert size *SD*^*^ | 94.3 | 54.3 |  |
| Strand specificity | Non-stranded | Non-stranded |  |
|  | Simulated (H) data | | |
|  | Yeast | Dog | Mouse |
| No. of fragments | 12,151,239 | 40,296,012 | 40,417,405 |
| *Max* Read length | 150 | 150 | 150 |
| Sequencing depth | 333.2 | 151.7 | 65.7 |
| Insert size *mean^*^* | 179.9 | 180.2 | 180.3 |
| Insert size *SD*^*^ | 45.2 | 45.1 | 45.1 |
| Strand specificity | Non-stranded | Non-stranded | Non-stranded |

^*^ The mean and standard deviation of the insert sizes were estimated using Burrows-Wheeler Aligner.

Table S3

*Definitions of contig categories*

| Categories | Definitions |
| --- | --- |
| Full-length | 1. Only **one** transcript assigned to the contig in interest. 2. The corresponding transcript only assigned to the contig in interest. 3. Both the contig and its corresponding transcript are **unique**. 4. Both *recovery* and *accuracy* of the global alignment (*contig*, *transcript*) $\geq$ 0.90 5. $-10\%\leq$ *difference in length* of (*contig*, *transcript*)$\leq10\%$ |
| Incompleteness | 1. Only **one** transcript assigned to the contig in interest. 2. The corresponding transcript only assigned to the contig in interest. 3. Both the contig and its corresponding transcript are **unique**. 4. The *accuracy* of the global alignment (*contig*, *transcript*) $\geq$ 0.90 5. *Difference in length* of (*contig*, *transcript*)$<-10\%$ |
| Over-extended | 1. Only **one** transcript assigned to the contig in interest. 2. The corresponding transcript only assigned to the contig in interest. 3. Both the contig and its corresponding transcript are **unique**. 4. The *recovery* of the global alignment (*contig*, *transcript*) $\geq$ 0.90 5. *Difference in length* of (*contig*, *transcript*)$> 10\%$ |
| Family-collapse | 1. All the transcripts that assigned to the contig in interest are in the same connected component. 2. All the corresponding transcript only assigned to the contig in interest. 3. The contig is **unique**. 4. All the transcripts that assigned to the contig are **not unique**. 5. There exists **at least one** global alignment (*contig*, *transcript*) that match the following criteria:    1. Both *recovery* and *accuracy* $\geq$ 0.90    2. $-10\%\leq$ *difference in length* $\leq10\%$ |
| Duplication | 1. Only **one** transcript assigned to the contig in interest. 2. The corresponding transcript is assigned to multiple contigs which are in the same connected component. 3. The contig is **not unique**. 4. The corresponding transcript is **unique**. 5. There exists **at least one** global alignment (*contig*, *transcript*) that match the following criteria:    1. Both *recovery* and *accuracy* $\geq$ 0.90    2. $-10\%\leq$ *difference in length* $\leq10\%$ |

Table S4

*Biological variability of reference transcripts.*

|  | Yeast | Dog | Mouse |
| --- | --- | --- | --- |
| No. of genes | 5,107 | 18,045 | 21,510 |
| No. of transcripts | 5,107 | 23,078 | 56,706 |
| No. of genes w/ single transcript | 5,107 | 13,713 | 8,071 |
| No. of transcripts per gene | 1.000 | 1.279 | 2.636 |
| Max number of transcripts for a gene | 1 | 7 | 47 |
| No. of unique transcripts | 4,806 | 15,299 | 16,134 |
| No. of connected components | 4,893 | 18,881 | 27,940 |
| Connected components size *mean* | 1.044 | 1.222 | 2.030 |
| Connected components size *max* | 62 | 26 | 163 |
| Maximum length | 14,733 | 105,543 | 123,179 |
| Total length | 8,512,860 | 62,014,091 | 143,577,796 |
| Average length | 1,666.90 | 2,687.15 | 2,531.97 |
| N50 | 1,944 | 3,776 | 3,523 |

Table S5

*Basic statistics for assembled contigs*

| Dataset | | Assembly | No. of contigs | No. of  unique contigs | No. of connected component | Connected component size *mean* | Connected component size *max* | Contigs  N50 |
| --- | --- | --- | --- | --- | --- | --- | --- | --- |
| Simulated | Yeast | rnaSPAdes | 5,620 | 5,550 | 5,582 | 1.007 | 4 | 1,575 |
|  |  | TransABySS | 5,443 | 5,264 | 5,325 | 1.022 | 9 | 1,652 |
|  |  | Trinity | 5,440 | 5,268 | 5,335 | 1.020 | 7 | 1,711 |
|  | Dog | rnaSPAdes | 12,189 | 11,736 | 11,953 | 1.020 | 7 | 2,797 |
|  |  | TransABySS | 14,239 | 11,030 | 12,177 | 1.169 | 30 | 2,687 |
|  |  | Trinity | 14,922 | 11,729 | 12,872 | 1.159 | 42 | 3,108 |
|  | Mouse | rnaSPAdes | 12,592 | 10,866 | 11,689 | 1.077 | 6 | 3,148 |
|  |  | TransABySS | 17,162 | 10,055 | 12,260 | 1.400 | 22 | 2,756 |
|  |  | Trinity | 16,320 | 10,276 | 12,423 | 1.314 | 24 | 3,295 |
| Experimental | Yeast | rnaSPAdes | 4,776 | 4,434 | 4,588 | 1.041 | 12 | 2,652 |
|  |  | TransABySS | 5,890 | 3,776 | 4,579 | 1.286 | 9 | 2,632 |
|  |  | Trinity | 5,506 | 4,143 | 4,704 | 1.170 | 22 | 2,634 |
|  | Dog | rnaSPAdes | 22,397 | 20,027 | 21,136 | 1.060 | 11 | 2,403 |
|  |  | TransABySS | 21,359 | 14,846 | 17,493 | 1.221 | 13 | 2,181 |
|  |  | Trinity | 21,607 | 17,652 | 19,278 | 1.121 | 21 | 2,141 |
|  | Mouse | rnaSPAdes | 24,522 | 18,064 | 20,792 | 1.179 | 18 | 3,186 |
|  |  | TransABySS | 37,840 | 15,511 | 21,995 | 1.720 | 24 | 2,413 |
|  |  | Trinity | 39,060 | 17,009 | 23,347 | 1.673 | 44 | 3,051 |
|  |  |  |  |  |  |  |  |  |
| Simulated (H) | Yeast | rnaSPAdes | 5,626 | 5,571 | 5,593 | 1.006 | 7 | 1,441 |
|  |  | TransABySS | 7,438 | 4,619 | 5,392 | 1.379 | 14 | 1,621 |
|  |  | Trinity | 5,443 | 5,293 | 5,346 | 1.018 | 10 | 1,713 |
|  | Dog | rnaSPAdes | 12,321 | 11,464 | 11,859 | 1.039 | 9 | 2,812 |
|  |  | TransABySS | 20,662 | 10,532 | 12,887 | 1.603 | 38 | 2,344 |
|  |  | Trinity | 15,297 | 11,866 | 13,089 | 1.169 | 38 | 3,018 |
|  | Mouse | rnaSPAdes | 12,185 | 10,453 | 11,265 | 1.082 | 7 | 3,223 |
|  |  | TransABySS | 19,939 | 10,031 | 12,641 | 1.577 | 37 | 2,539 |
|  |  | Trinity | 16,805 | 10,340 | 12,618 | 1.332 | 61 | 3,330 |
| Experimental (H) | Yeast | rnaSPAdes | 4,042 | 3,575 | 3,791 | 1.066 | 8 | 3,584 |
|  |  | TransABySS | 7,447 | 2,285 | 3,855 | 1.932 | 15 | 3,019 |
|  |  | Trinity | 4,942 | 3,218 | 3,888 | 1.271 | 11 | 3,764 |
|  | Dog | rnaSPAdes | 22,919 | 19,906 | 21,283 | 1.077 | 12 | 2,702 |
|  |  | TransABySS | 25,375 | 14,263 | 18,439 | 1.376 | 19 | 2,540 |
|  |  | Trinity | 23,358 | 16,883 | 19,393 | 1.204 | 36 | 2,724 |

Table S6

*Number of contigs for contig categories*

| Dataset | | Assembly | Full-length | Over- extension | Incomp-leteness | Family-collapse | Duplication |
| --- | --- | --- | --- | --- | --- | --- | --- |
| Simulated | Yeast | rnaSPAdes | 2,831 | 11 | 564 | 52 | 15 |
|  |  | TransABySS | 3,175 | 5 | 482 | 43 | 69 |
|  |  | Trinity | 3,306 | 5 | 382 | 51 | 34 |
|  | Dog | rnaSPAdes | 3,546 | 70 | 827 | 1,469 | 22 |
|  |  | TransABySS | 3,340 | 36 | 868 | 1,056 | 460 |
|  |  | Trinity | 3,402 | 61 | 944 | 1,090 | 288 |
|  | Mouse | rnaSPAdes | 1,638 | 98 | 253 | 3,076 | 14 |
|  |  | TransABySS | 1,492 | 71 | 293 | 2,288 | 339 |
|  |  | Trinity | 1,597 | 92 | 287 | 2,431 | 142 |
| Experimental | Yeast | rnaSPAdes | 689 | 1,151 | 132 | 7 | 13 |
|  |  | TransABySS | 657 | 868 | 153 | 5 | 229 |
|  |  | Trinity | 525 | 1,067 | 141 | 5 | 119 |
|  | Dog | rnaSPAdes | 441 | 1,385 | 2,077 | 209 | 120 |
|  |  | TransABySS | 320 | 804 | 1,832 | 145 | 348 |
|  |  | Trinity | 368 | 973 | 1,963 | 149 | 148 |
|  | Mouse | rnaSPAdes | 1,544 | 231 | 585 | 2,353 | 626 |
|  |  | TransABySS | 972 | 84 | 545 | 988 | 2,813 |
|  |  | Trinity | 1,176 | 111 | 585 | 1,103 | 1,733 |
|  |  |  |  |  |  |  |  |
| Simulated (H) | Yeast | rnaSPAdes | 2,360 | 9 | 718 | 44 | 6 |
|  |  | TransABySS | 2,558 | 4 | 411 | 27 | 2,254 |
|  |  | Trinity | 3,338 | 8 | 368 | 48 | 10 |
|  | Dog | rnaSPAdes | 3,357 | 55 | 824 | 1,319 | 135 |
|  |  | TransABySS | 2,829 | 29 | 882 | 808 | 3,225 |
|  |  | Trinity | 3,535 | 64 | 945 | 1,102 | 277 |
|  | Mouse | rnaSPAdes | 1,590 | 77 | 235 | 3,041 | 37 |
|  |  | TransABySS | 1,364 | 63 | 290 | 2,106 | 841 |
|  |  | Trinity | 1,598 | 93 | 291 | 2,405 | 177 |
| Experimental (H) | Yeast | rnaSPAdes | 353 | 1,045 | 24 | 9 | 11 |
|  |  | TransABySS | 258 | 596 | 38 | 3 | 422 |
|  |  | Trinity | 286 | 892 | 54 | 5 | 65 |
|  | Dog | rnaSPAdes | 503 | 1,628 | 1,890 | 238 | 170 |
|  |  | TransABySS | 370 | 943 | 1,553 | 174 | 615 |
|  |  | Trinity | 446 | 1,244 | 1,775 | 214 | 271 |

Table S7

*Coefficient of variation for estimated expression*

| Dataset | | Assembly | Kallisto | RSEM | Salmon |
| --- | --- | --- | --- | --- | --- |
| Simulated | Yeast | rnaSPAdes | 191.16 | 187.55 | 191.14 |
|  |  | TransABySS | 180.63 | 180.45 | 181.16 |
|  |  | Trinity | 200.84 | 193.51 | 203.89 |
|  | Dog | rnaSPAdes | 272.60 | 269.47 | 272.48 |
|  |  | TransABySS | 282.84 | 284.87 | 282.71 |
|  |  | Trinity | 292.82 | 294.68 | 292.46 |
|  | Mouse | rnaSPAdes | 324.82 | 328.24 | 324.68 |
|  |  | TransABySS | 369.80 | 372.62 | 369.94 |
|  |  | Trinity | 398.59 | 398.44 | 399.18 |
| Experimental | Yeast | rnaSPAdes | 376.85 | 363.72 | 377.17 |
|  |  | TransABySS | 403.34 | 411.45 | 404.00 |
|  |  | Trinity | 362.39 | 367.83 | 361.99 |
|  | Dog | rnaSPAdes | 578.62 | 587.95 | 579.05 |
|  |  | TransABySS | 614.56 | 616.80 | 616.64 |
|  |  | Trinity | 595.46 | 604.05 | 594.75 |
|  | Mouse | rnaSPAdes | 1139.32 | 1090.57 | 1132.04 |
|  |  | TransABySS | 1294.32 | 1317.81 | 1296.66 |
|  |  | Trinity | 1279.79 | 1330.67 | 1314.44 |
