# Supplementary material for: Effect of de novo transcriptome assembly on transcript quantification
Source: Sci Rep. 2019 Jun 5;9:8304. doi: 10.1038/s41598-019-44499-3 (PMC6549443; doi:10.1038/s41598-019-44499-3)
Supplement: Supplementary file 2 [file 41598_2019_44499_MOESM2_ESM.pdf]

Effect of *de novo* transcriptome assembly on transcript quantification

Ping-Han Hsieh, Yen-Jen Oyang and Chien-Yu Chen

Supplementary File 2

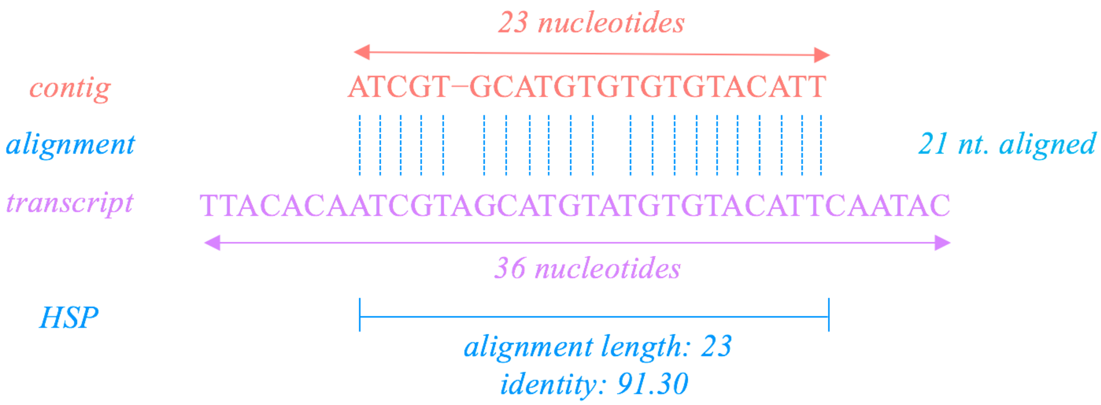

$$recovery = \frac{23 \times 91.30}{36} = 0.583 \qquad accuracy = \frac{23 \times 91.30}{23} = 0.913$$

Fig. S1: Recovery and Accuracy.

The diagram illustrates a global alignment between a contig and a transcript. The blue dot lines between sequences represent the matched nucleotides suggested by BLASTN. Based on the global alignment, the recovery is defined as the proportion of nucleotides on transcript that are reconstructed by the contig, while the accuracy is defined as the proportion of nucleotides correctly matched on the contig. Note that these metrics are used to describe the global alignments between contigs and transcripts instead of contig or transcript sequences.

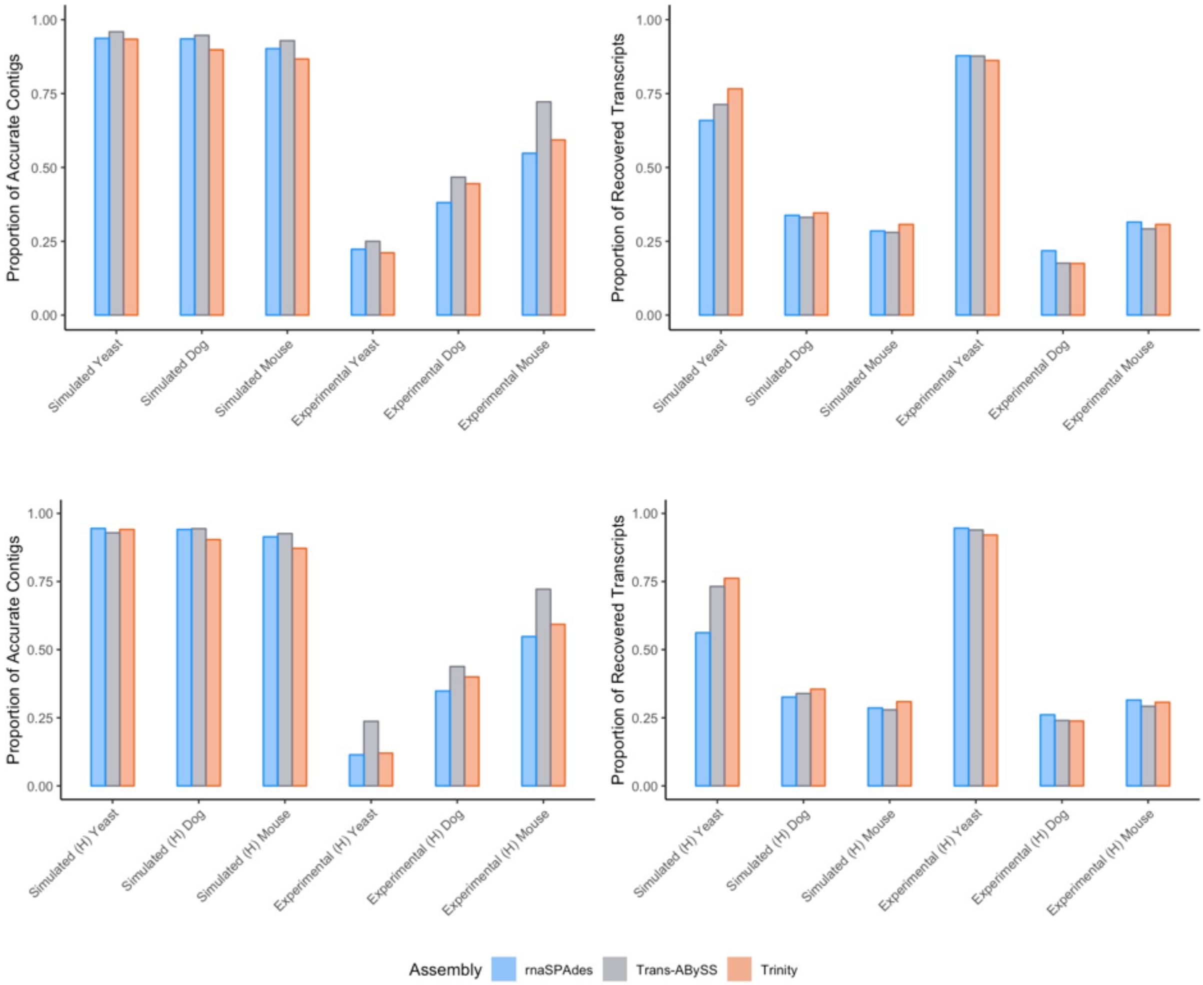

Fig. S2: Proportion of Accurate Contigs and Recovered Transcripts

The bar plots illustrate the proportion of accurate contigs (contig aligned with at least one transcript that shows *accuracy* $\geq$ 90) and recovered transcripts (transcript aligned with at least one contig that shows *recover*y $\geq$ 90). In general, the proportion of recovered transcripts is significantly higher for yeast dataset. It appears to be more difficult for the assemblers to properly reconstruct the transcriptome for sequences with higher complexity. Moreover, the proportion of correct contigs for Trans-ABySS is higher in nearly all the datasets and the proportion of recovered transcripts for Trinity is higher in the simulated datasets. However, the performance between different assemblers shows only marginal difference.

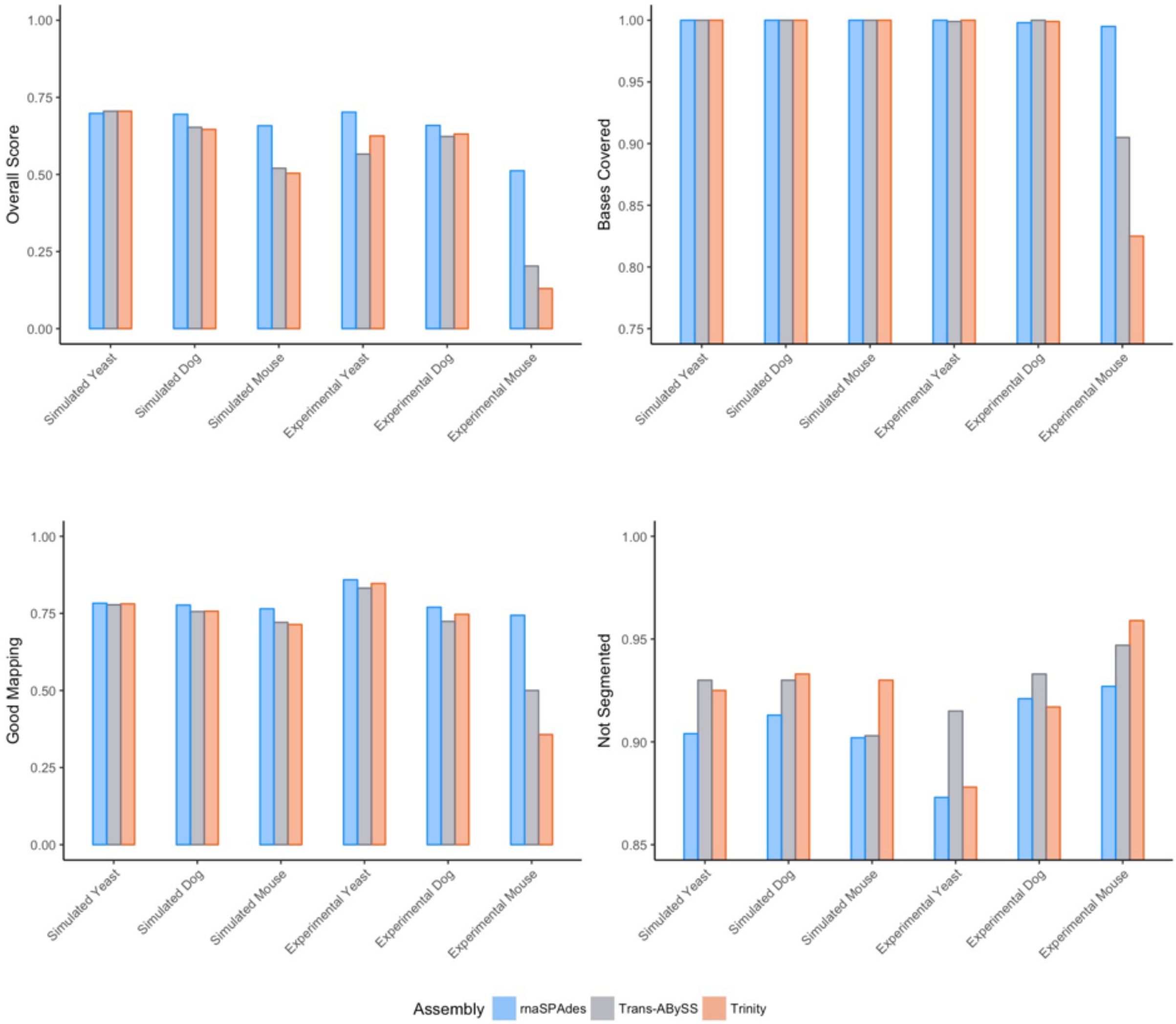

Fig. S3-1: Median of TransRate Scores for Simulated and Experimental Datasets

The bar plots illustrate the median of TransRate scores for assembled contigs constructed from simulated and experimental datasets. In general, the *overall TransRate scores* are higher for the contigs constructed by rnaSPAdes. However, the median of TransRate scores of *Bases Covere*d, *Good* and *Not Segmented* varied greatly across different dataset, therefore, it is hard to conclude which assembler outperformed the others based on these metrics.

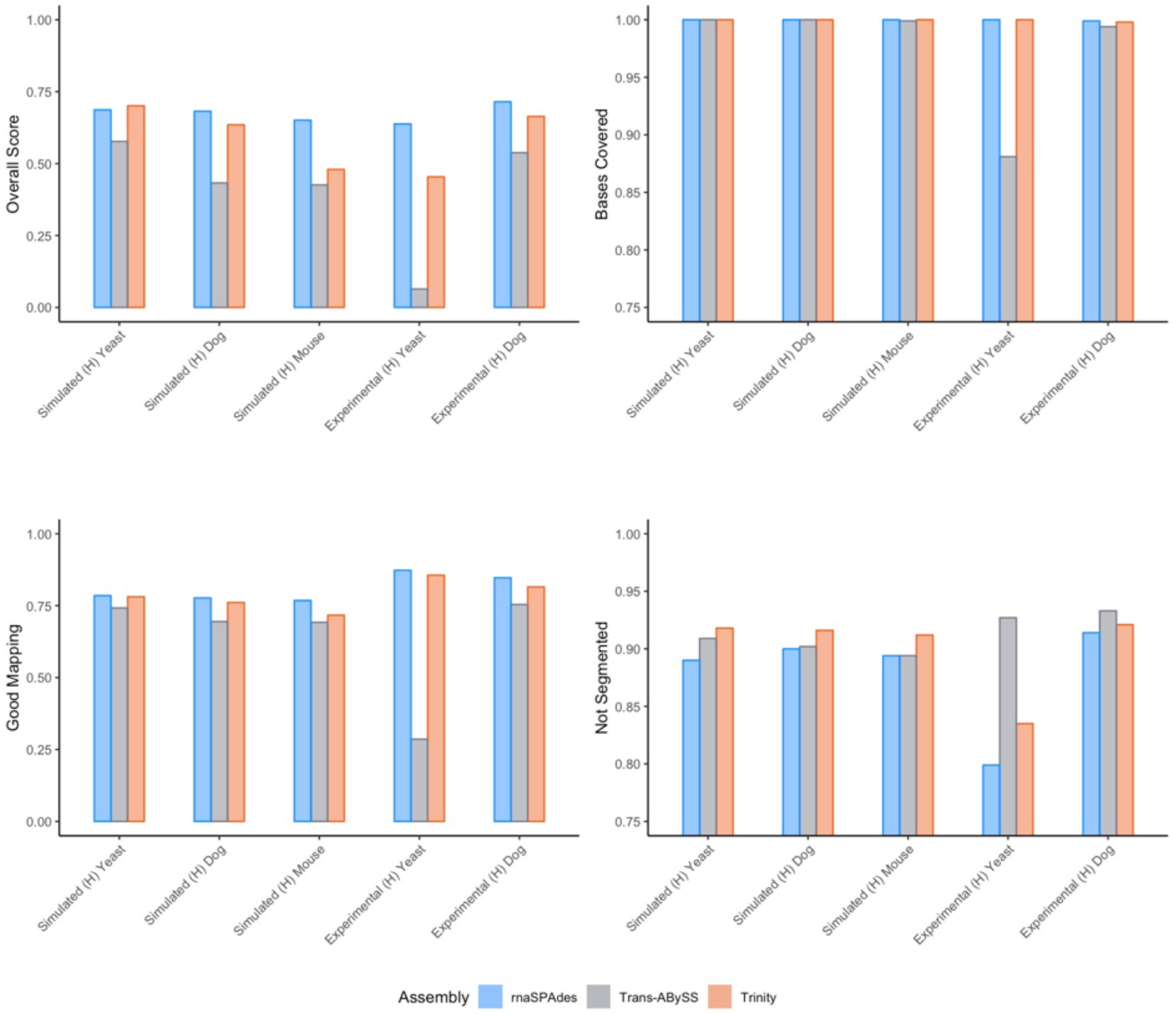

Fig. S3-2: Median of TransRate Scores for Simulated (H) and Experimental (H) Datasets
The bar plots illustrate the median of TransRate scores for assembled contigs constructed from simulated (H) and experimental (H) datasets. In general, the *overall TransRate scores* are higher for the contigs constructed by rnaSPAdes. However, the median of TransRate scores of *Bases Covere*d, *Good* and *Not Segmented* varied greatly across different dataset, therefore, it is hard to conclude which assembler outperformed the others based on these metrics.

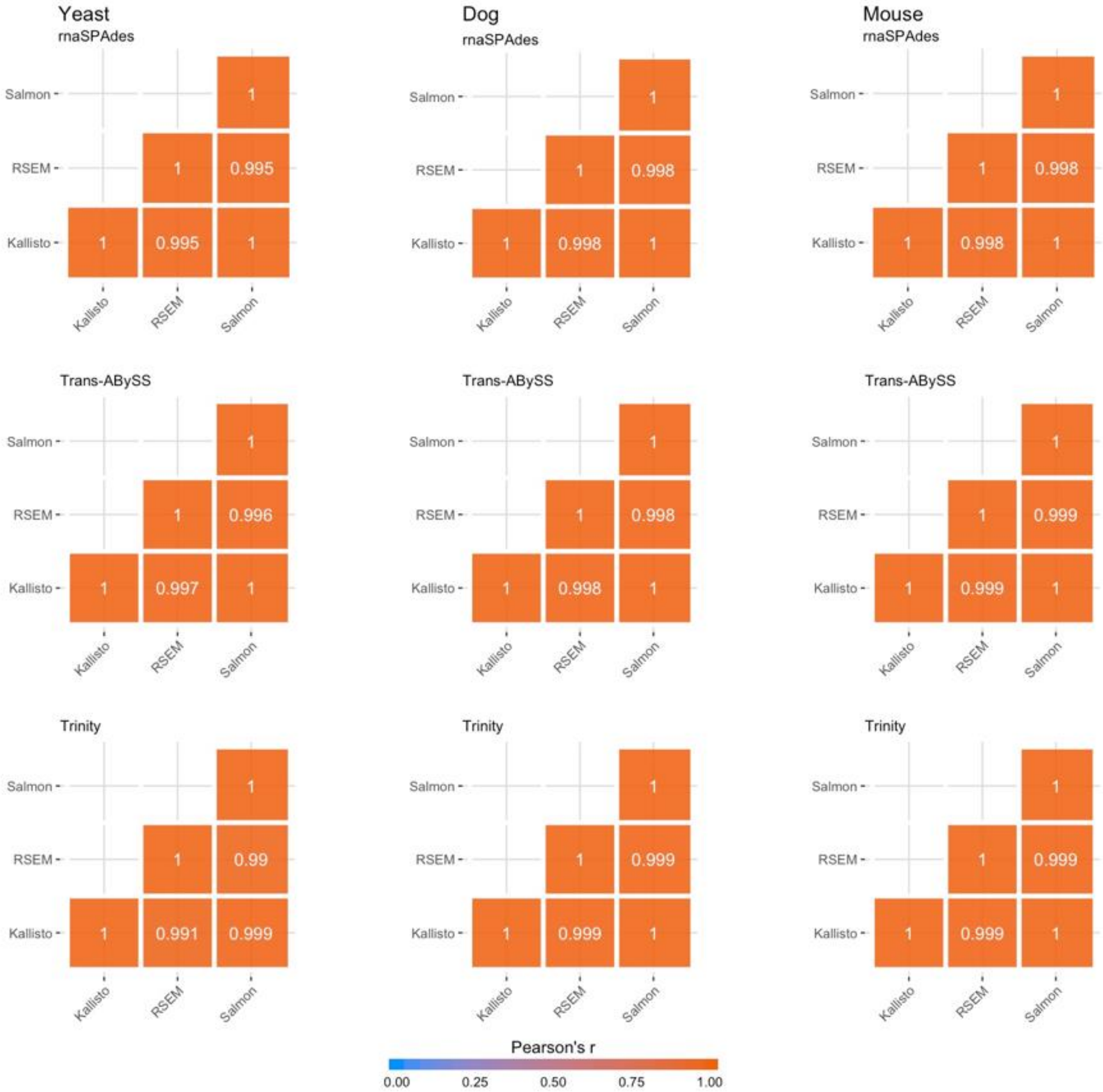

Fig. S4-1: Pearson's Correlation Coefficient between Quantifiers (Simulated)

The correlation matrices illustrate the Pearson's correlation coefficient between the estimation made by any of the two quantifiers. The matrices in the left column are the results drawn from yeast dataset, in the middle column are the result from dog dataset and in the right column from mouse dataset. The matrices in the first row are the estimation based on rnaSPAdes assembly, the second row on Trans-ABySS assembly, and the third row on Trinity. In general, the correlation matrices show high consistency for the estimation made by quantifiers on simulated datasets.

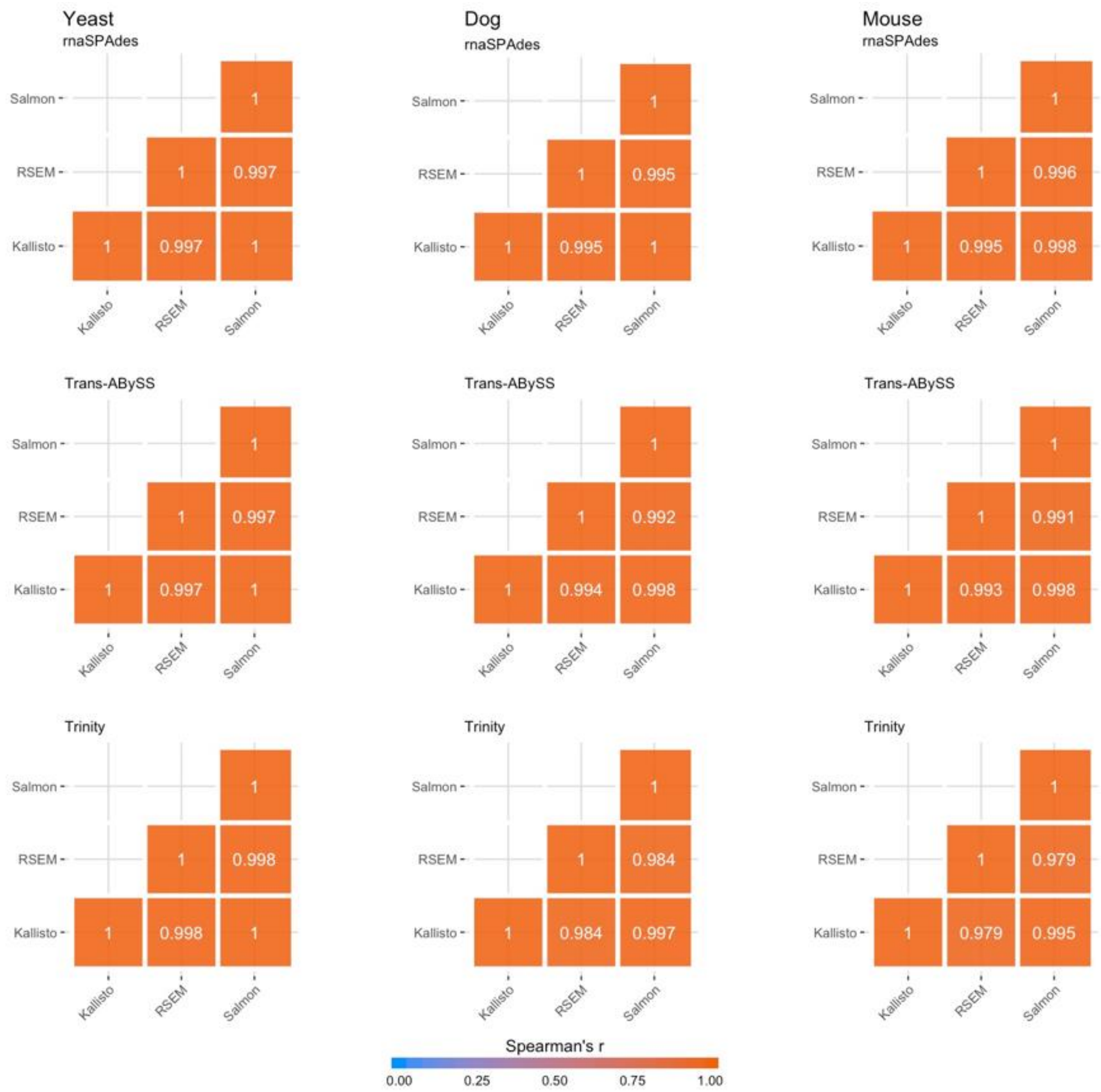

Fig. S4-2: Spearman's Correlation Coefficient between Quantifiers (Simulated)

The correlation matrices illustrate the Spearman's correlation coefficient between the estimation made by any of the two quantifiers. The matrices in the left column are the results drawn from yeast dataset, in the middle column are the result from dog dataset and in the right column from mouse dataset. The matrices in the first row are the estimation based on rnaSPAdes assembly, the second row on Trans-ABySS assembly, and the third row on Trinity. In general, the correlation matrices show high consistency for the estimation made by quantifiers on simulated datasets.

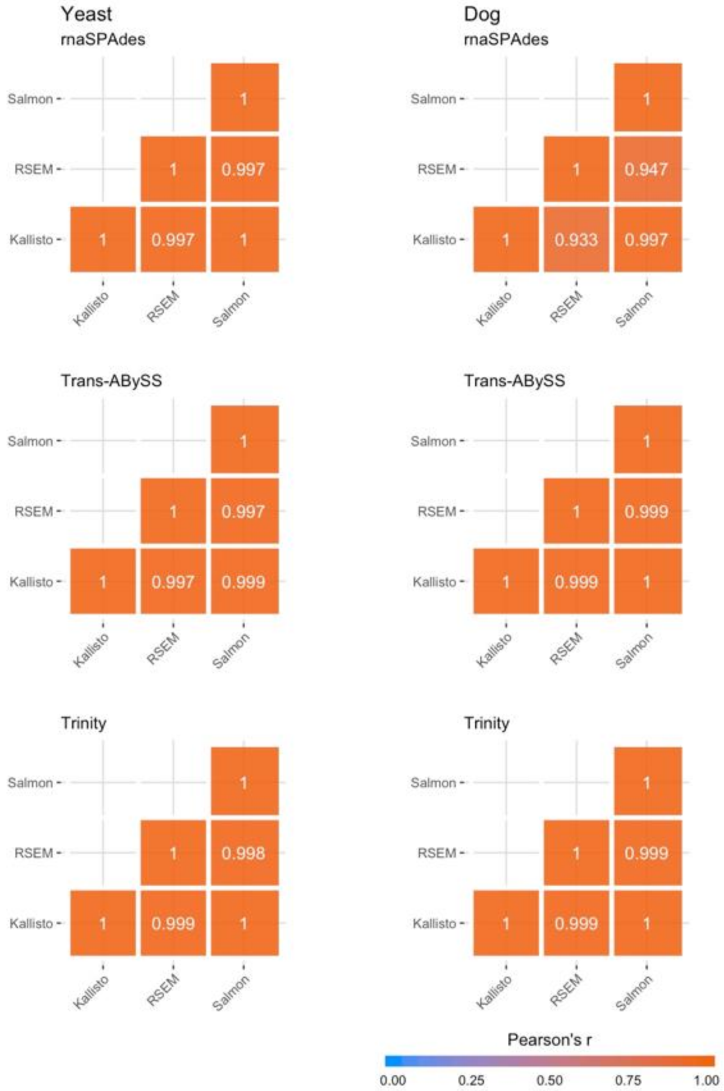

Fig. S4-3: Pearson's Correlation Coefficient between Quantifiers (Simulated H)

The correlation matrices illustrate the Pearson's correlation coefficient between the estimation made by any of the two quantifiers. The matrices in the left column are the results drawn from yeast dataset, in the middle column are the result from dog dataset and in the right column from mouse dataset. The matrices in the first row are the estimation based on rnaSPAdes assembly, the second row on Trans-ABySS assembly, and the third row on Trinity. In general, the correlation matrices show high consistency for the estimation made by quantifiers on simulated datasets.

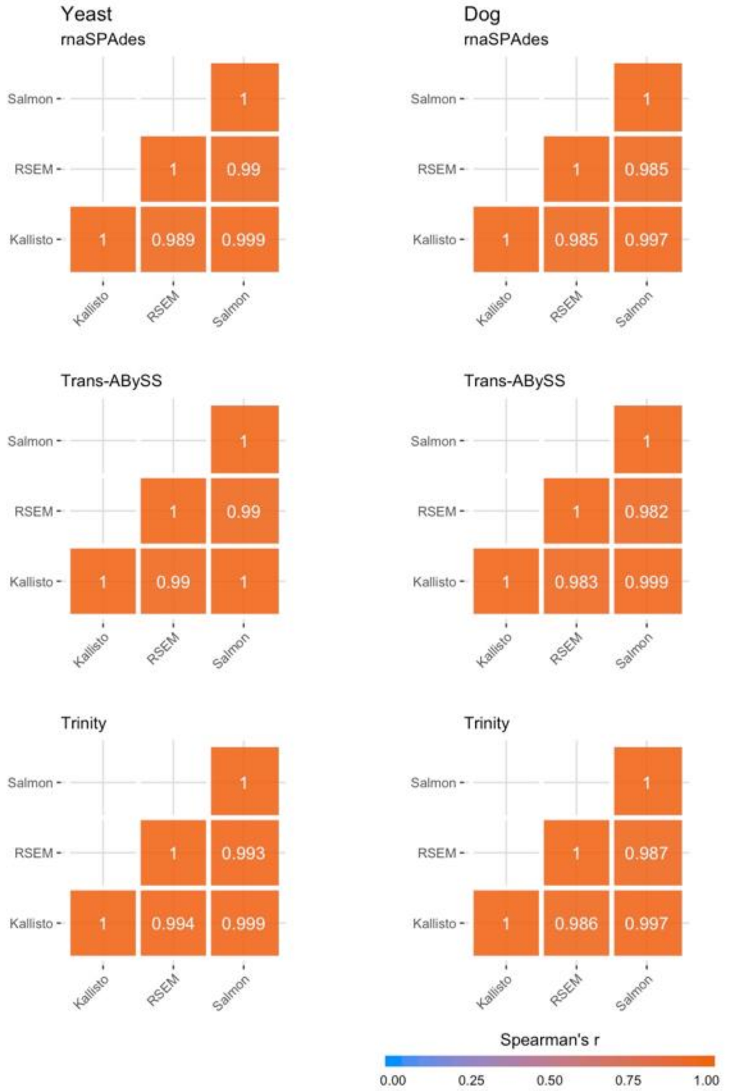

Fig. S4-4: Spearman's Correlation Coefficient between Quantifiers (Simulated H)

The correlation matrices illustrate the Spearman's correlation coefficient between the estimation made by any of the two quantifiers. The matrices in the left column are the results drawn from yeast dataset, in the middle column are the result from dog dataset and in the right column from mouse dataset. The matrices in the first row are the estimation based on rnaSPAdes assembly, the second row on Trans-ABySS assembly, and the third row on Trinity. In general, the correlation matrices show high consistency for the estimation made by quantifiers on simulated datasets.

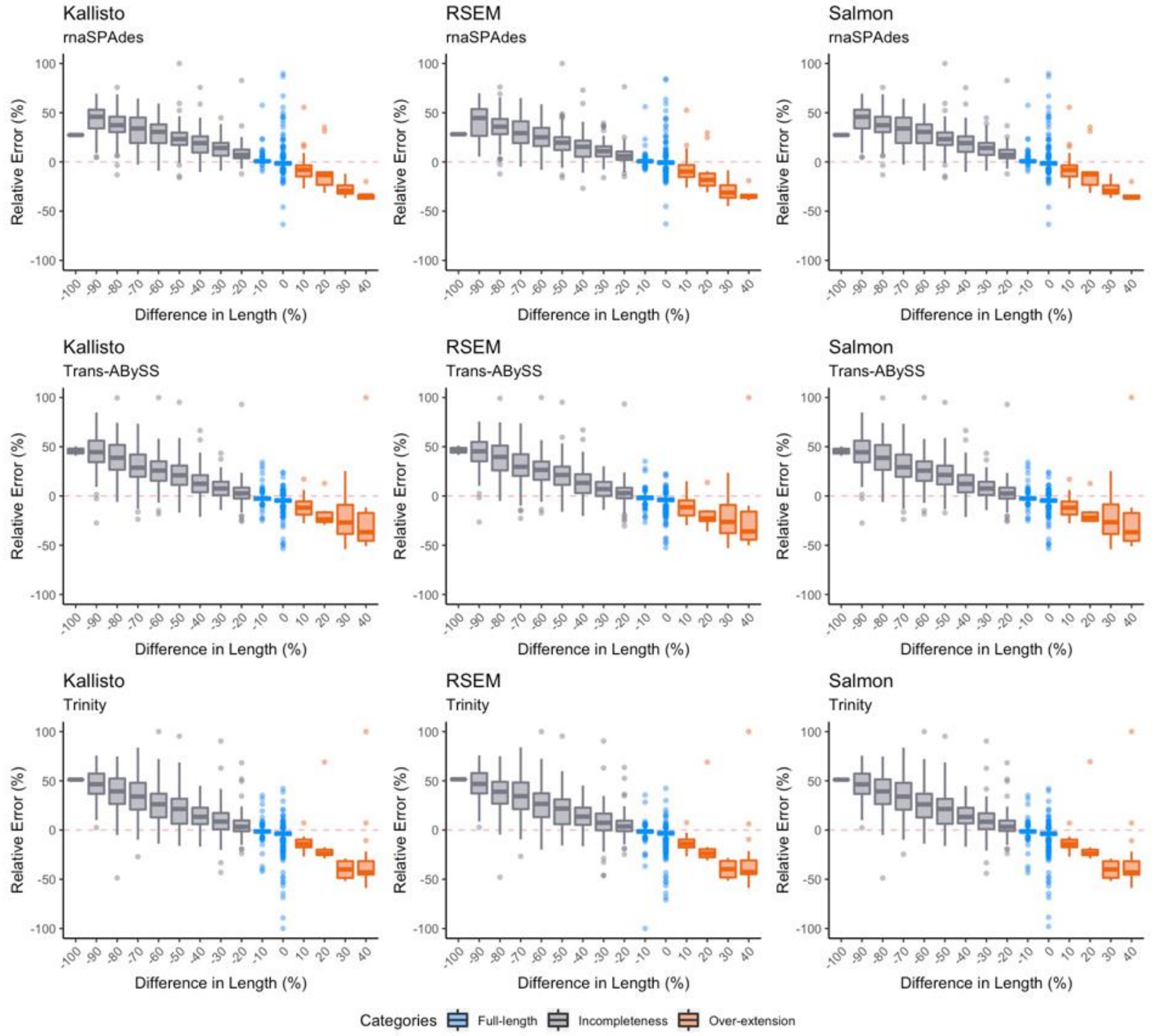

Fig. S5: Quantification Errors for Unique Sequences for Simulated (H) Dog Dataset

The box plots illustrate the relative quantification errors for unique contigs on the simulated (H) dog datasets. The contigs are grouped by the extent of assembly completeness, and the numbers on the X-axis represent the lower bound of differences in length. For instance, the contigs located on -10 means that the percentage of difference in length is in the range of $[-10, 0)$. The data is color-coded based on the contig categories. The box plots in the top row are based on the rnaSPAdes assembly, the second row on Trans-ABySS assembly, and the third row on Trinity. The first column are the results based on Kallisto, while the second and third column depict the results of RSEM and Salmon respectively. The box plots suggest that the estimation made on full-length contigs yield the smallest relative errors, while the incomplete contigs show over-estimation and over-extended contigs show under-estimation on quantification.

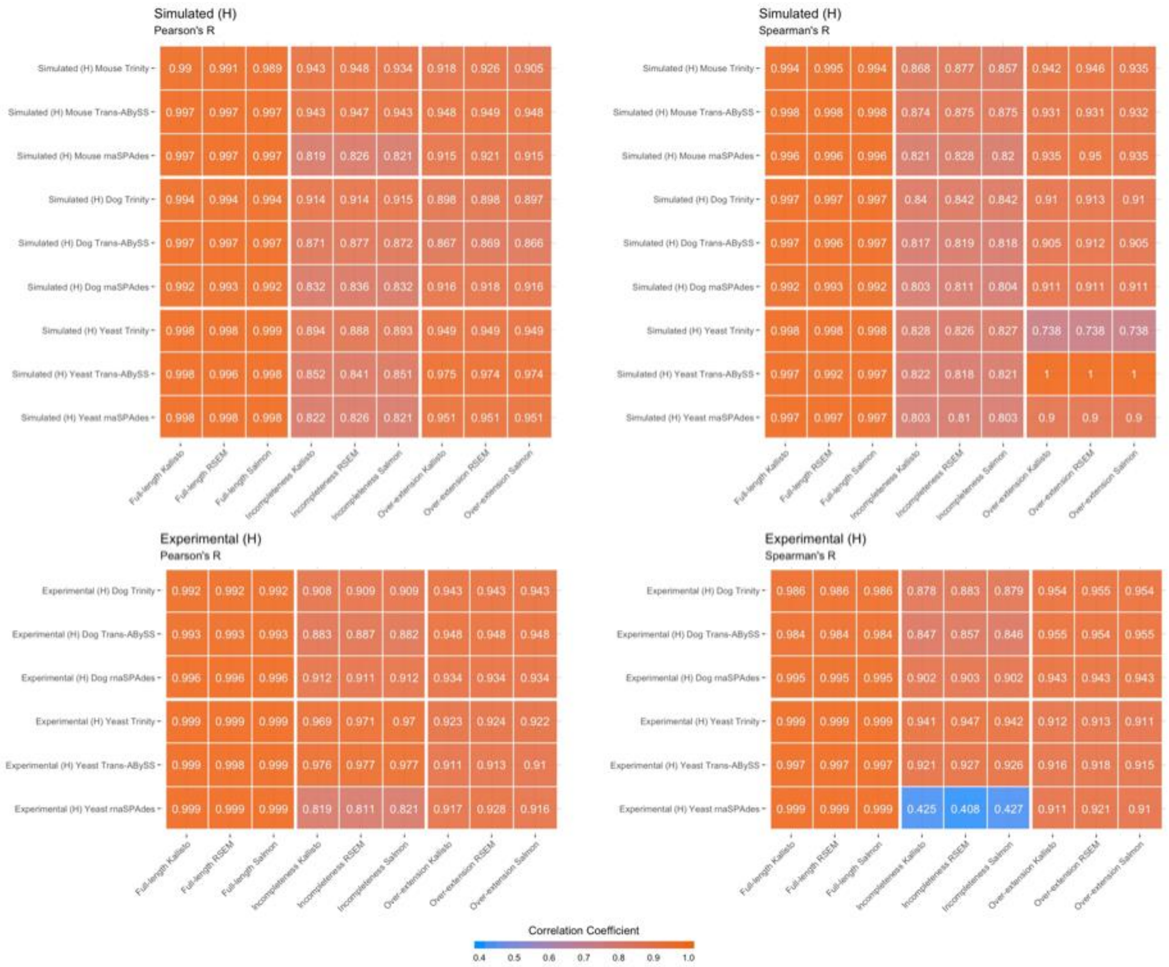

Fig. S6: Correlation Coefficients for Unique Sequences of (H) Datasets

The figures illustrate the Pearson's and Spearman's correlation coefficients between estimated abundance and ground truth expression of Simulated (H) and Experimental (H) Datasets. In general, the estimation based on full-length contigs have considerably high correlation with the ground truth expression of corresponding transcripts. In contrast, the incomplete and over-extended contigs show relatively lower correlation coefficients.

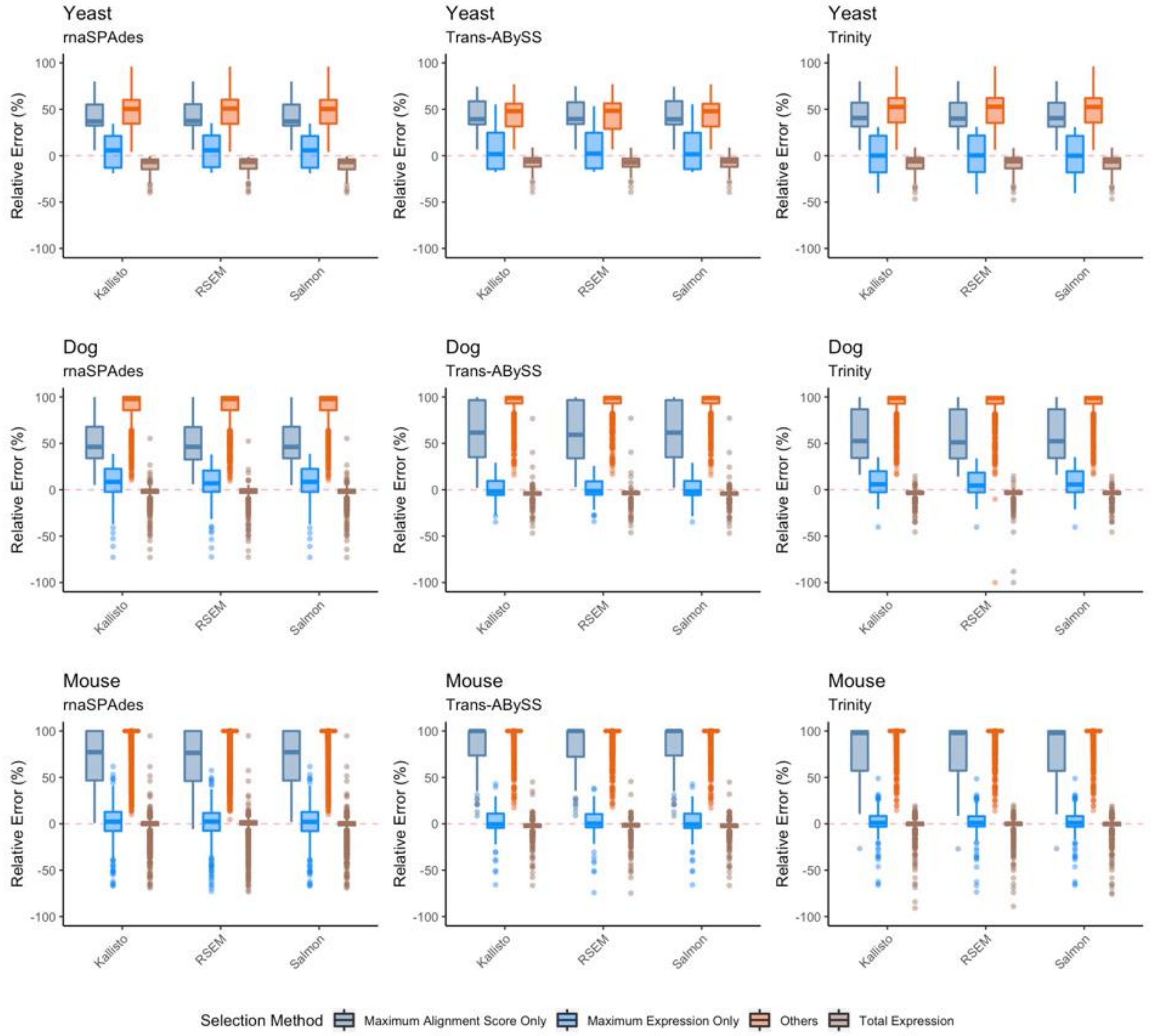

Fig. S7-1: Box Plots for the Relative Errors of Family-collapse Contigs (Simulated)

The box plots illustrated the relative quantification errors for family-collapse contigs of simulated datasets. Since there are multiple transcripts that correspond to one contig, we categorized the expression of corresponding transcripts into (1) transcripts with the maximum alignment score (but the expression is not the highest), (2) transcripts with the highest expression (but the alignment score is not the highest), (3) others and (4) total expression of the connected components of the transcripts. The box plots in the left column are based on the rnaSPAdes assembly, the second column on Trans-ABySS assembly, and the third column on Trinity. The first row is the result from the yeast dataset, while the second and third rows depicted that of dog and mouse, respectively. Overall, the box plots suggested that the estimation made on family-collapse contigs is closest to the total expression of the connected component of transcripts.

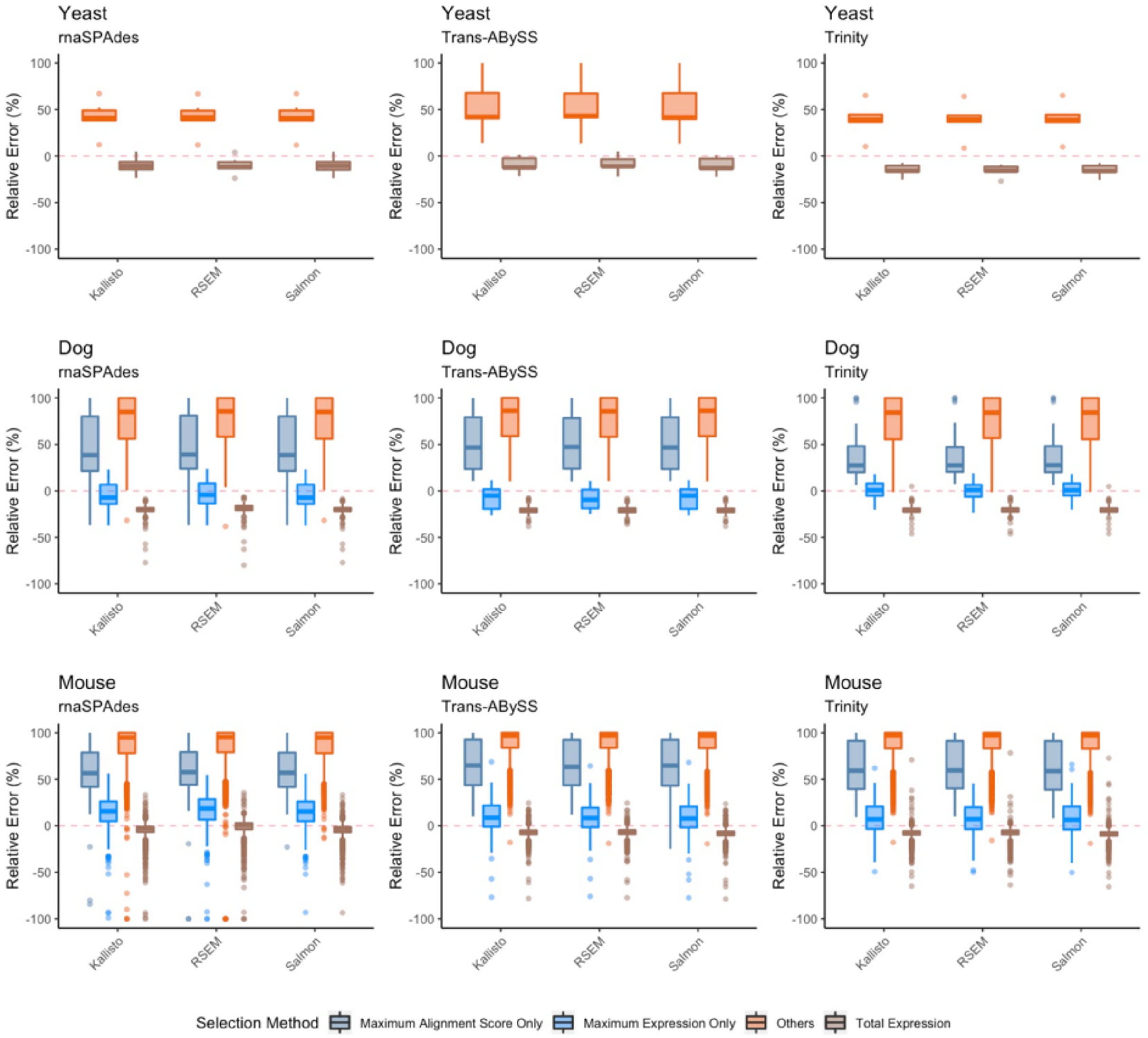

Fig. S7-2: Box Plots for the Relative Errors of Family-collapse Contigs (Experimental)

The box plots illustrated the relative quantification errors for family-collapse contigs of experimental datasets. Since there are multiple transcripts that correspond to one contig, we categorize the expression of corresponding transcripts into (1) transcripts with the maximum alignment score (but the expression is not the highest), (2) transcripts with the highest expression (but the alignment score is not the highest), (3) others and (4) total expression of connected component of the transcripts. The box plots in the left column are based on the rnaSPAdes assembly, the second column on Trans-ABySS assembly, and the third column on Trinity. The first row is the result from the yeast dataset, while the second and third rows depicted that of dog and mouse respectively. Overall, the box plots suggested that the estimation made on family-collapse contigs is closest to the total expression of the connected component of transcripts.

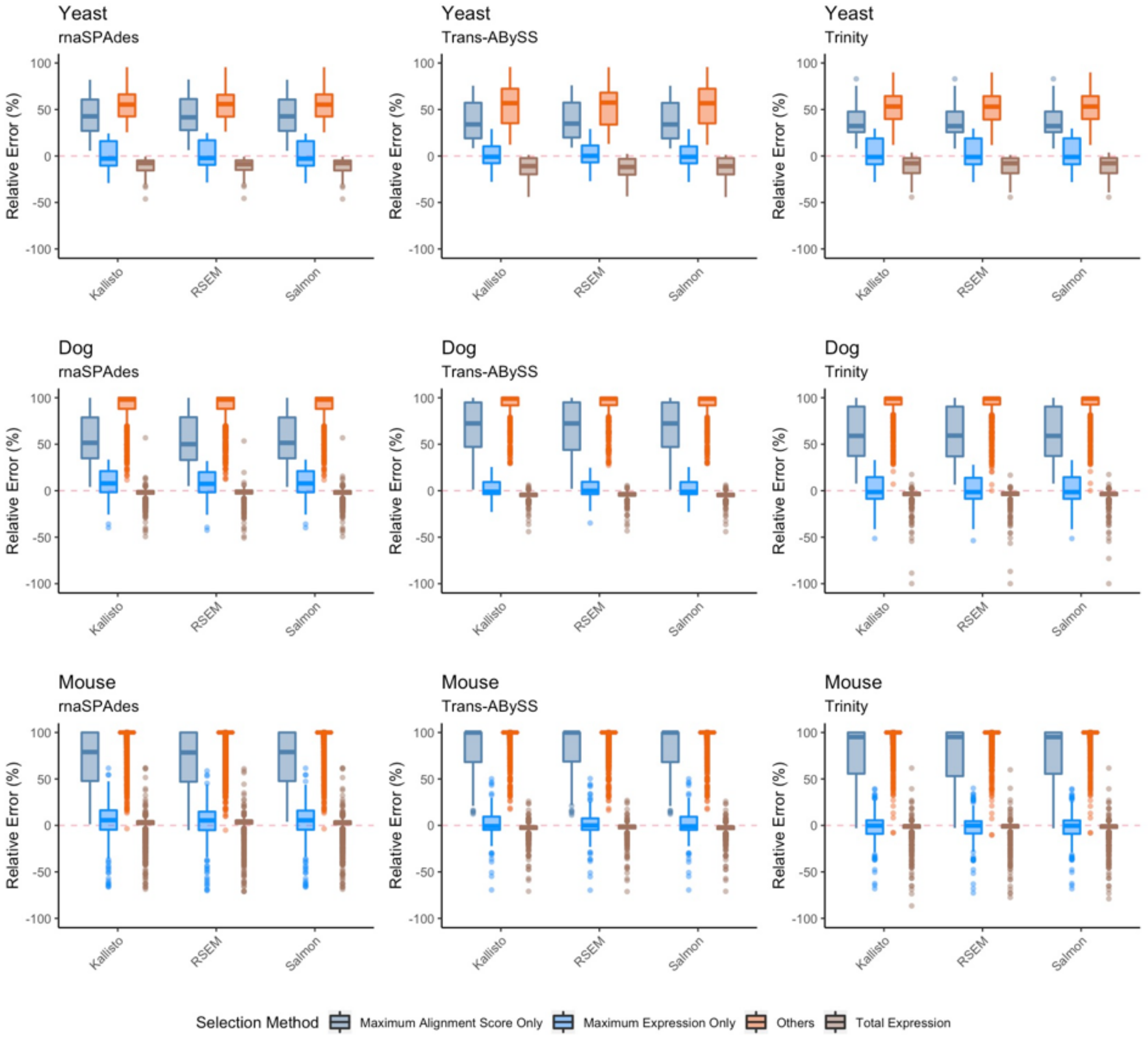

Fig. S7-3: Box Plots for the Relative Errors of Family-collapse Contigs (Simulated H)

The box plots illustrated the relative quantification errors for family-collapse contigs of simulated (H) datasets. Since there are multiple transcripts that correspond to one contig, we categorized the expression of corresponding transcripts into (1) transcripts with the maximum alignment score (but the expression is not the highest), (2) transcripts with the highest expression (but the alignment score is not the highest), (3) others and (4) total expression of the connected components of the transcripts. The box plots in the left column are based on the rnaSPAdes assembly, the second column on Trans-ABySS assembly, and the third column on Trinity. The first row is the result from the yeast dataset, while the second and third rows depicted that of dog and mouse, respectively. Overall, the box plots suggested that the estimation made on family-collapse contigs is closest to the total expression of the connected component of transcripts.

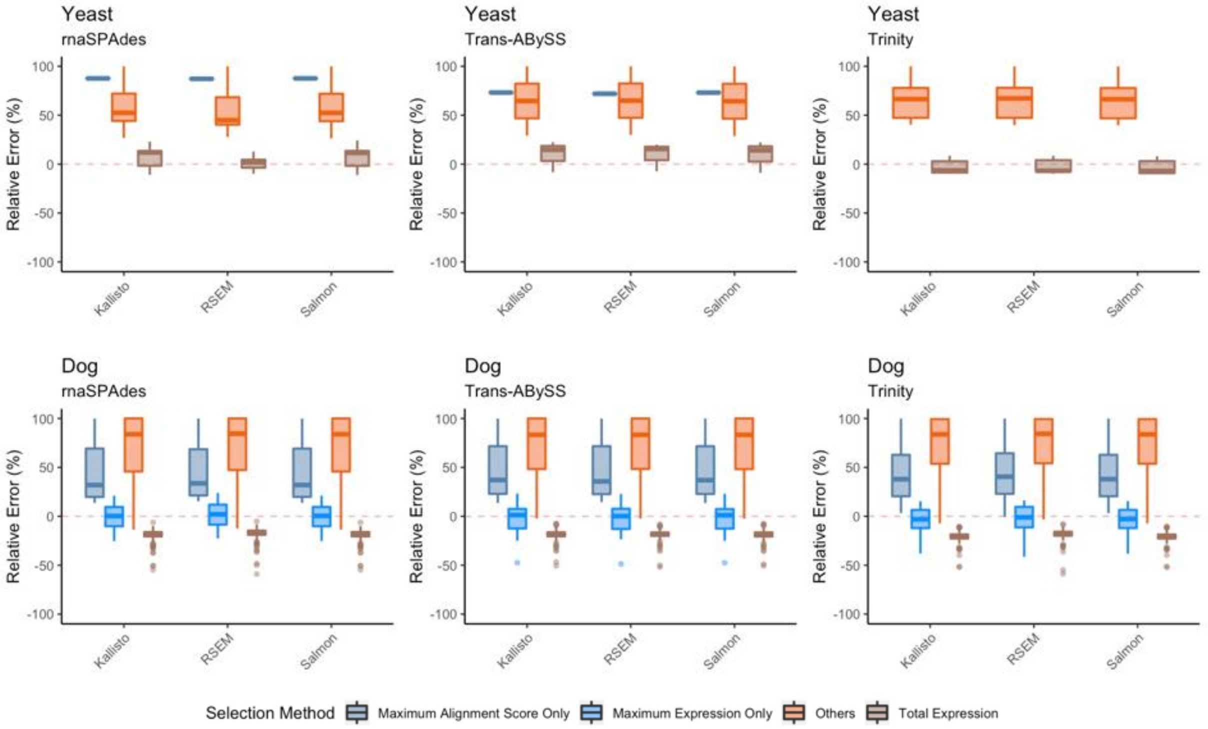

Fig. S7-4: Box Plots for the Relative Errors of Family-collapse Contigs (Experimental H) The box plots illustrated the relative quantification errors for family-collapse contigs of experimental (H) datasets. Since there are multiple transcripts that correspond to one contig, we categorize the expression of corresponding transcripts into (1) transcripts with the maximum alignment score (but the expression is not the highest), (2) transcripts with the highest expression (but the alignment score is not the highest), (3) others and (4) total expression of connected component of the transcripts. The box plots in the left column are based on the rnaSPAdes assembly, the second column on Trans-ABySS assembly, and the third column on Trinity. The first row is the result from the yeast dataset, while the second and third rows depicted that of dog and mouse respectively. Overall, the box plots suggested that the estimation made on family-collapse contigs is closest to the total expression of the connected component of transcripts.

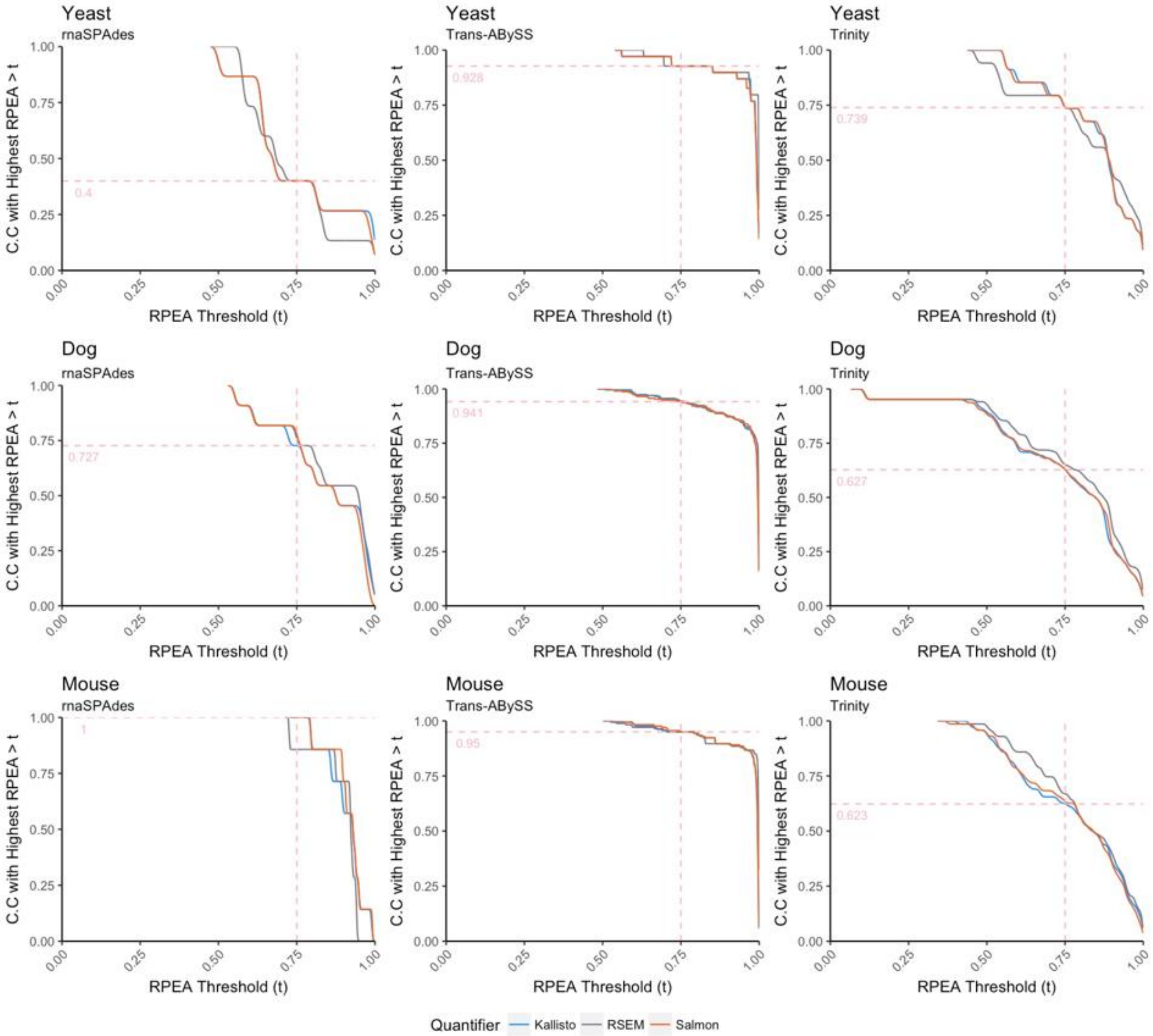

Fig. S8-1: Proportion of the Duplicated Connected Component with Highest Read Proportion (Simulated)

The line graphs illustrate the proportion of connected components for duplicated contigs of simulated dataset. The X-axis is the threshold of RPEA (t) while the Y-axis is the proportion of connected component with the highest RPEA > t. By this mean, we are allowed to examine how quantifiers allocate the RNA reads for duplicated contigs. Based on our result, most of the connected components have at least one contig that contribute over 0.75 of its expression, which suggest that the quantifiers tend to allocate most of the RNA reads to a single contig in the connected component instead of distributing evenly.

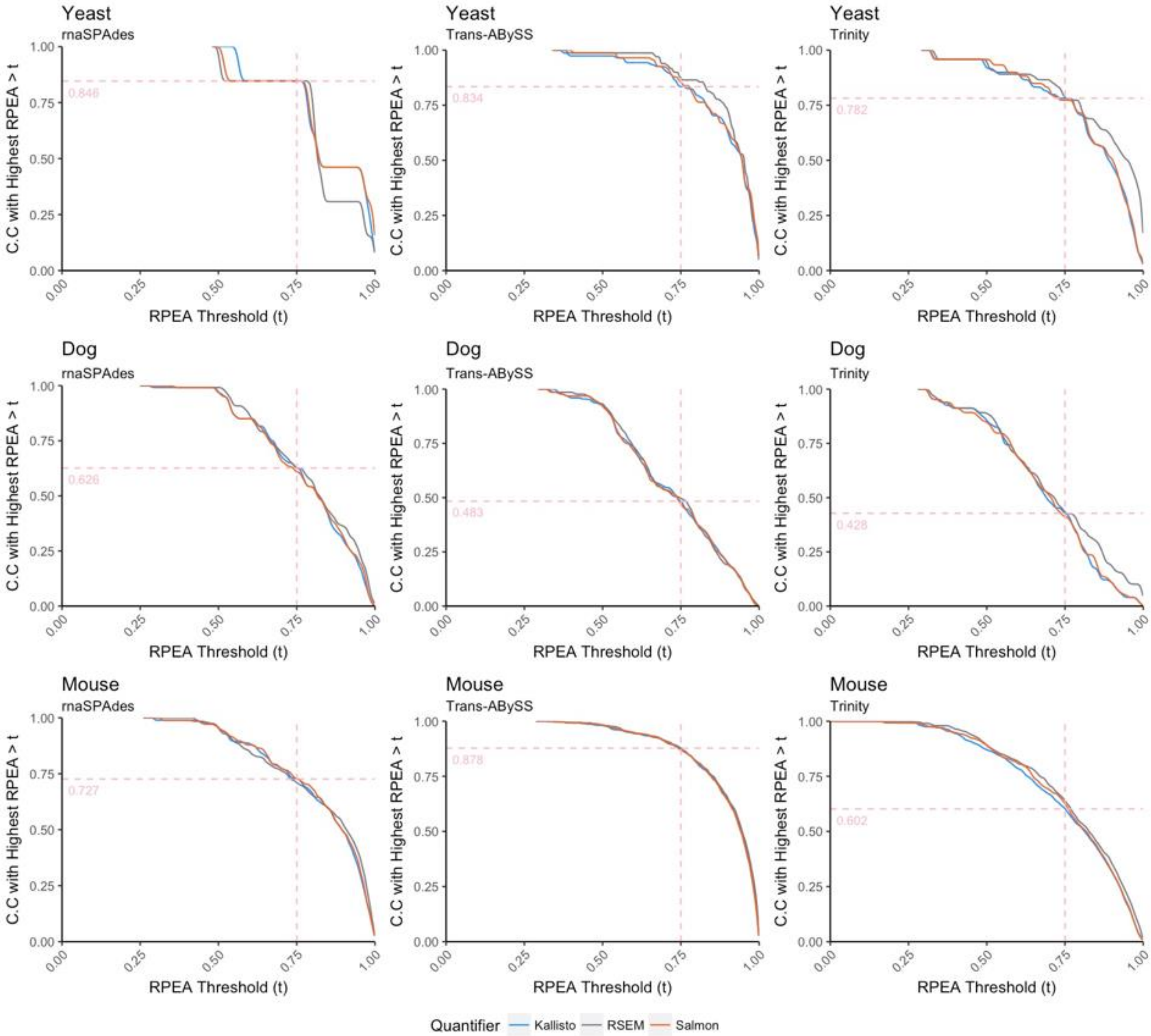

Fig. S8-2: Proportion of the Duplicated Connected Component with Highest Read Proportion (Experimental)

The line graphs illustrate the proportion of connected components for duplicated contigs of experimental dataset. The X-axis is the threshold of RPEA (*t*) while the Y-axis is the proportion of connected component with the highest RPEA > *t*. By this mean, we are allowed to examine how quantifiers allocate the RNA reads for duplicated contigs. Based on our result, most of the connected components have at least one contig that contribute over 0.75 of its expression, which suggest that the quantifiers tend to allocate most of the RNA reads to a single contig in the connected component instead of distributing evenly.

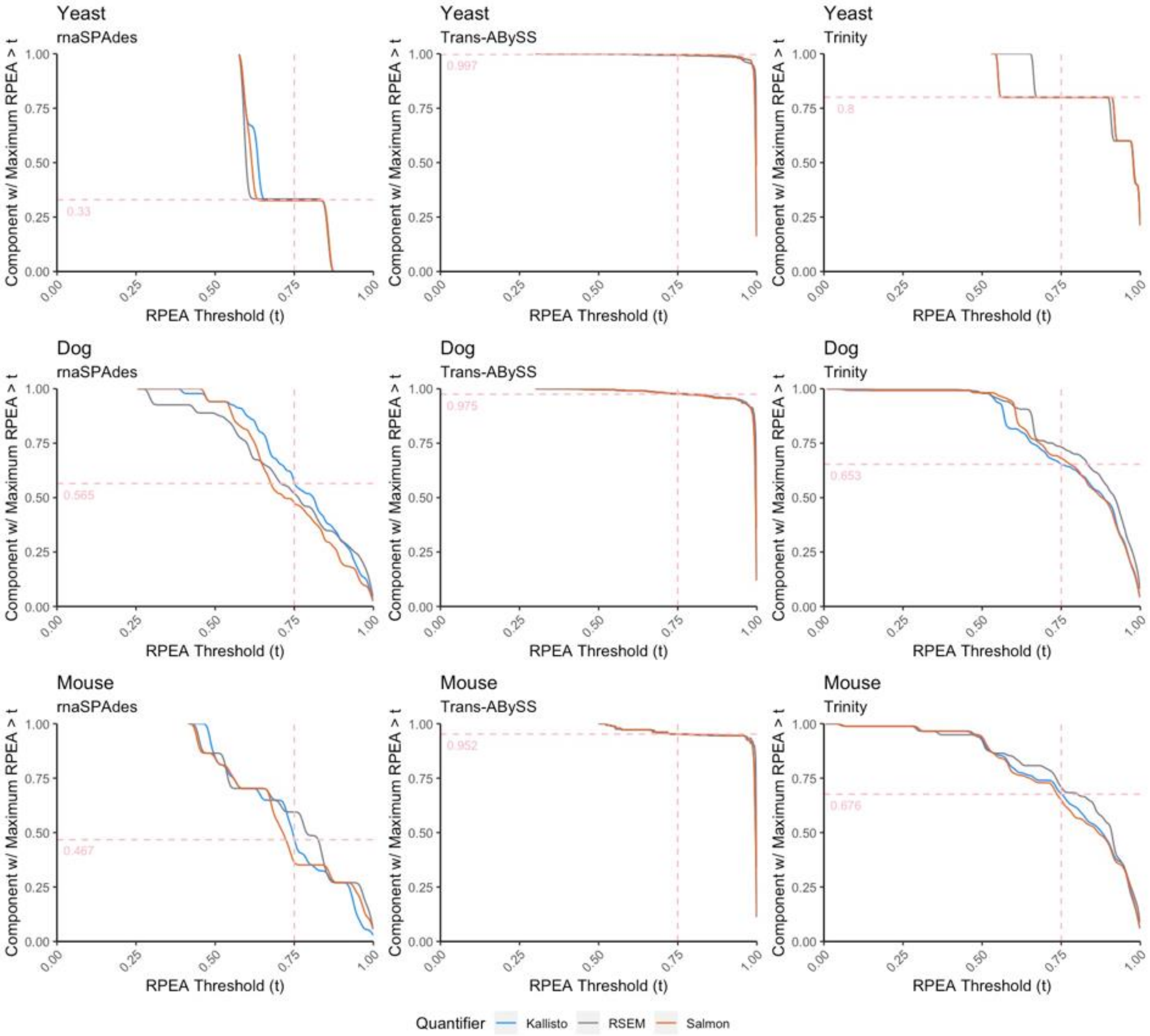

Fig. S8-3: Proportion of the Duplicated Connected Component with Highest Read Proportion (Simulated H)

The line graphs illustrate the proportion of connected components for duplicated contigs of simulated (H) dataset. The X-axis is the threshold of RPEA (t) while the Y-axis is the proportion of connected component with the highest RPEA > t. By this mean, we are allowed to examine how quantifiers allocate the RNA reads for duplicated contigs. Based on our result, most of the connected components have at least one contig that contribute over 0.75 of its expression, which suggest that the quantifiers tend to allocate most of the RNA reads to a single contig in the connected component instead of distributing evenly.

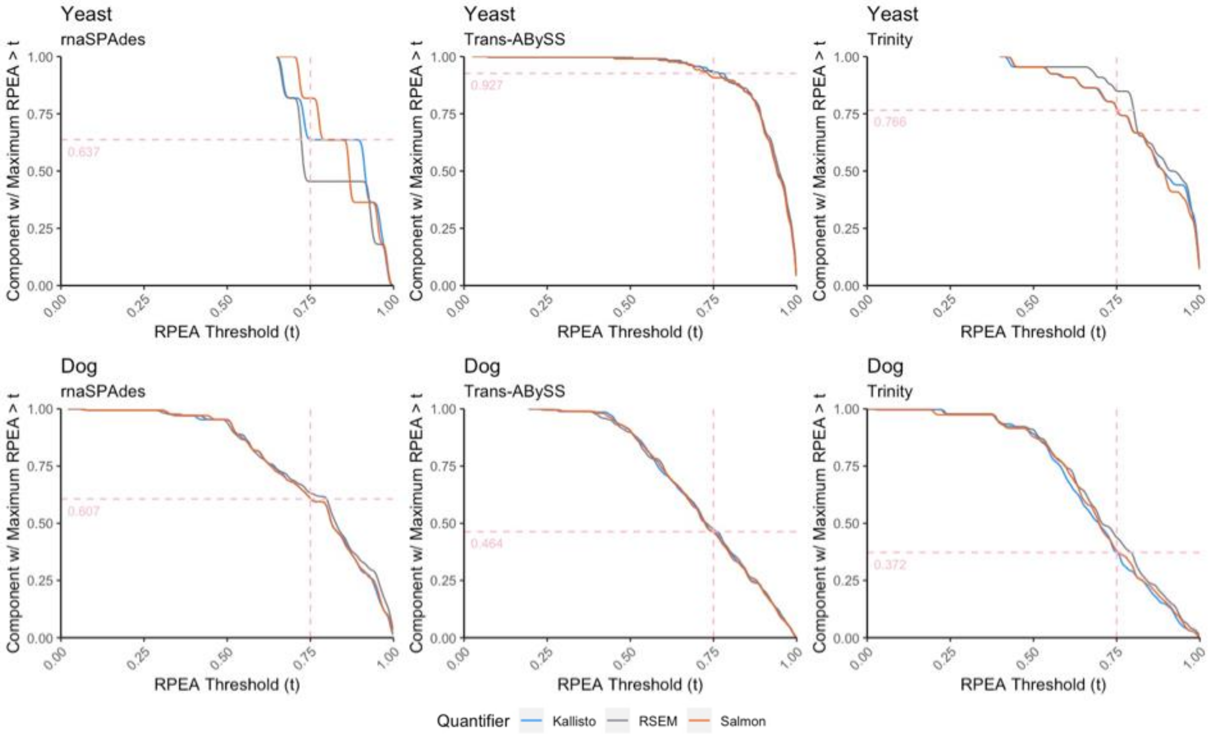

Fig. S8-4: Proportion of the Duplicated Connected Component with Highest Read Proportion (Experimental H)

The line graphs illustrate the proportion of connected components for duplicated contigs of experimental dataset. The X-axis is the threshold of RPEA (t) while the Y-axis is the proportion of connected component with the highest RPEA > t. By this mean, we are allowed to examine how quantifiers allocate the RNA reads for duplicated contigs. Based on our result, most of the connected components have at least one contig that contribute over 0.75 of its expression, which suggest that the quantifiers tend to allocate most of the RNA reads to a single contig in the connected component instead of distributing evenly.

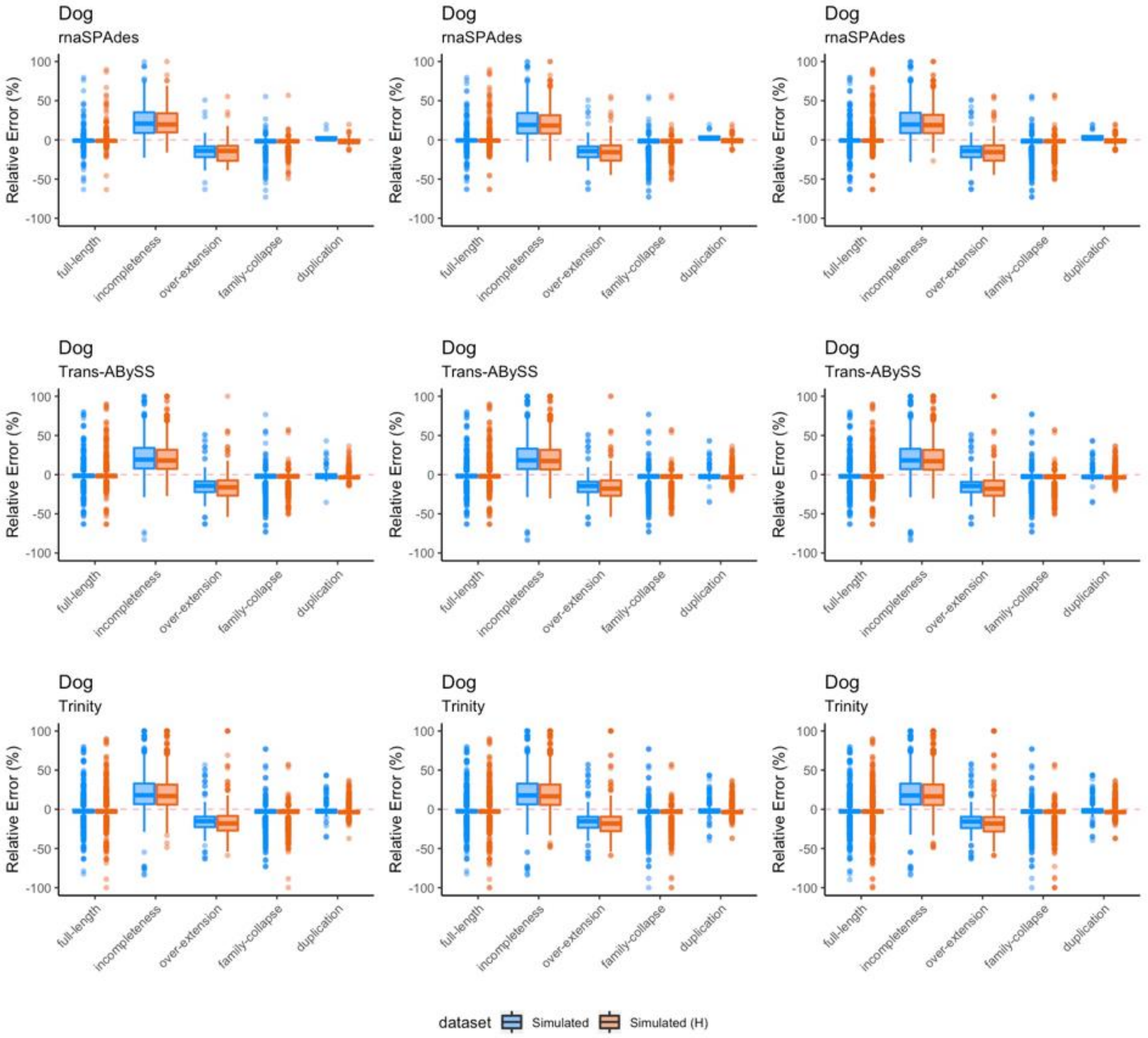

Fig. S9: Comparison of Quantification Errors for Each Category between Datasets

The box plots illustrate the relative quantification errors for each category for the assembled contigs on the simulated dog datasets (also with higher sequencing depth). The results from simulated dog datasets are shown in blue color, while the results from higher sequencing depth are shown in red color. The box plots in the top row are based on the rnaSPAdes assembly, the second row on Trans-ABySS assembly, and the third row on Trinity. The first column are the results based on Kallisto, while the second and third column depict the results of RSEM and Salmon respectively. The box plots suggest that the estimation made on full-length contigs yield the smallest relative errors, while the incomplete contigs show over-estimation and over-extended contigs show under-estimation on quantification.
